# Student Perceptions of Key Terminology Tests in Dental Physiology Education: A Single-Institution Evaluation

**DOI:** 10.3390/dj14070461

**Published:** 2026-07-22

**Authors:** Tatsuko Yokota, Tomoko Matsunaga, Nobuhiko Hatanaka, Hiroki Toyoda

**Affiliations:** Department of Physiology, School of Dentistry, Aichi Gakuin University, Nagoya 464-8650, Japan

**Keywords:** dental physiology, terminology tests, learning motivation, CBT preparation, glossary use

## Abstract

**Objective**: This study evaluated the educational value and motivational impact of unit-based Key Terminology Tests, combined with a dedicated glossary, in a second-year dental physiology course designed to support preparation for computer-based testing (CBT) and the Japanese National Dentist Examination. **Methods**: Seventy-six second-year dental students completed periodic terminology tests aligned with lecture units (50 items per test, 25 min each). Bonus points were awarded for scores of ≥45/50. At the end of the semester, students completed an anonymous questionnaire assessing perceived test burden (scope, duration, and number of items), preparation time, clarity and use of the glossary, perceived contribution to lecture comprehension, and the motivational impact of bonus points. Quantitative data were summarized descriptively, and free-text responses were analyzed thematically. **Results**: Most students rated the test scope and number of questions as appropriate (50.0% and 67.1%, respectively), although 48.7% perceived the scope as somewhat excessive or excessive, and 35.6% felt the time limit was short or somewhat short. The glossary improved lecture comprehension for 68.4% of respondents, and bonus points enhanced learning motivation for 53.9%. In addition, 86.9% reported using the glossary at least occasionally during routine study. **Conclusions**: Students perceived the terminology test system as supportive of lecture comprehension, perceived learning support, and sustained learning motivation, suggesting that it may serve as a useful preparatory approach for CBT and the National Dentist Examination.

## 1. Introduction

Physiology constitutes a foundational pillar of dental education, providing essential knowledge for understanding oral function, systemic interactions, and the pathophysiological mechanisms that underlie clinical dentistry [1,2]. Mastery of physiological concepts is particularly critical for second-year dental students, who are transitioning from basic biomedical sciences to preclinical training. At this stage, students are required to assimilate an extensive body of specialized terminology related to neuromuscular control of mastication, salivary gland function, sensory and nociceptive pathways, and homeostatic regulation—domains that directly inform the interpretation of dental diseases and clinical decision-making [3,4]. However, acquiring this terminology poses substantial cognitive demands, and many students struggle to develop both accurate recall and conceptual understanding.

The challenge is amplified by the sheer volume of terminology, often encompassing several hundred terms per course, which can promote surface-level memorization rather than deep learning [5]. This issue becomes particularly salient in high-stakes assessments such as computer-based testing (CBT) and the Japanese National Dentist Examination, where students must not only recall terminology but also apply physiological principles to clinical scenarios [6]. Consequently, there is a growing need for instructional strategies that support systematic terminology acquisition while reinforcing conceptual comprehension.

A substantial body of educational research highlights the effectiveness of formative assessment strategies—including low-stakes testing, spaced repetition, and frequent retrieval practice—in strengthening long-term retention through the testing effect [7,8,9]. These approaches are widely recognized as evidence-based methods for promoting durable learning [10]. Within dental education, terminology-focused interventions have demonstrated benefits when they are closely aligned with lecture content, mechanistic explanations, and clinical relevance [11,12]. Despite these advances, relatively few reports have described terminology-learning interventions that are explicitly integrated with lecture units and linked to competencies relevant to licensure examinations. Furthermore, maintaining student engagement in demanding preclinical curricula requires thoughtful motivational design, including the incorporation of extrinsic incentives such as bonus points to encourage sustained participation [13,14].

To address these challenges, the present study evaluated a structured “Key Terminology Test System” implemented in second-year dental physiology lectures. The system incorporated a custom Dental Physiology Key Terminology Glossary containing definitions, mechanistic explanations, and diagrams relevant to core physiological concepts. Periodic terminology tests were administered throughout the semester. Each test consisted of 50 questions completed within 25 min and was based on approximately 65 key terms included in the glossary for each lecture unit. Students who achieved the bonus-point threshold (≥45 correct answers) received bonus points as an extrinsic motivator to reinforce consistent study habits.

Although terminology-learning interventions have been explored in both dental and medical education, many prior studies have emphasized perceived usefulness rather than objectively measured learning gains. The present study therefore focused on evaluating students’ perceptions of this terminology test system as a tool for foundational learning. Because the evaluation relies exclusively on post-intervention self-reported perceptions, it does not assess objective educational outcomes such as examination performance or long-term retention.

The aim of this study was to examine the system’s perceived impact on lecture comprehension, study habits, and motivation, and to identify areas for improved alignment with CBT and national examination requirements. An end-of-semester survey assessed perceived burden, preparation time, glossary utility, motivational effects, and usage patterns, supplemented by qualitative comments that provide deeper insight into student experiences. Within the context of Japanese dental education, such structured terminology-learning interventions may contribute to strengthening foundational physiological understanding and enhancing students’ perceived readiness for licensure examinations.

## 2. Methods

### 2.1. Participants

Participants were second-year dental students enrolled in a compulsory physiology course at the School of Dentistry, Aichi Gakuin University, during the 2025 academic year. The course is positioned in the preclinical phase of the curriculum and is designed to provide foundational knowledge in systemic and oral physiology prior to clinical training. Of the 80 students registered for the course, 76 completed the questionnaire, yielding a response rate of 95.0%. No exclusion criteria were applied, and all respondents who returned fully completed questionnaires were included in the analysis.

Participation in the survey component of the study was strictly voluntary. At the time of questionnaire administration, students received a standardized written and verbal explanation outlining the purpose of the educational evaluation, the anonymous nature of data collection, and the absence of any academic consequences associated with participation or non-participation. They were explicitly informed that their decision to participate would not influence their grades, course evaluation, or relationship with faculty members, and that no coercion, undue pressure, or incentives were involved. Questionnaires were completed in the classroom immediately after the final terminology test session, and responses were collected without any identifying information to promote candid feedback.

This study was conducted as a single-institution, cross-sectional educational evaluation based solely on post-intervention student perceptions. No pre-intervention baseline data, longitudinal follow-up, or control-group comparisons were obtained, and no objective measures such as examination scores or long-term retention indices were linked to individual questionnaire responses. The questionnaire was originally administered as part of an internal educational quality assurance process, and academic publication was not planned at the time of data collection. The data were collected anonymously or were subsequently anonymized to ensure participant confidentiality. At a later stage, after the decision was made to use the data for research purposes, informed consent for academic dissemination was obtained from all participating students through a written consent form.

The study was conducted in accordance with the ethical principles of the Declaration of Helsinki. The research protocol, including the retrospective academic use of anonymized educational data and the consent procedures, was reviewed and approved by the Institutional Review Board of the School of Dentistry, Aichi Gakuin University (Approval No. 787; approved on 15 April 2026). The approval explicitly covered the use of these data for scholarly publication and ensured that the study met institutional and national ethical standards for educational research.

### 2.2. Intervention

The educational intervention consisted of a structured terminology-learning system embedded within the existing physiology course. A Dental Physiology Key Terminology Glossary was developed specifically for this course, compiling approximately 65 key terms for each lecture unit. Lecture units covered major domains of dental physiology, including neuromuscular control of mastication, salivary secretion, sensory and nociceptive pathways, cardiovascular and respiratory physiology, and homeostatic regulation relevant to oral health. The target number of 65 terms per unit was selected to ensure comprehensive coverage of core physiological concepts considered essential for understanding orofacial function and for preparing students for computer-based testing (CBT)-style assessments.

Each glossary entry included three structured components: (1) a concise definition emphasizing the core concept; (2) a brief mechanistic explanation highlighting underlying physiological processes; and (3) illustrative diagrams, where appropriate, to emphasize dental and clinical relevance. The glossary was distributed to students in printed and/or digital format at the beginning of each lecture unit, and students were encouraged to use it both during lectures and in their independent study.

Of the approximately 65 glossary terms per unit, 50 were selected for each terminology test. The selection process aimed to balance breadth of coverage with feasibility under time constraints, prioritizing terms that represented central physiological mechanisms and those considered highly relevant for CBT and the Japanese National Dentist Examination. Selecting 50 items allowed students to practice rapid retrieval of a broad range of foundational concepts under time constraints similar to those encountered in CBT examinations.

Each test consisted of 50 fill-in-the-blank items and was completed within a 25-min time limit. The items were presented as short statements describing the definition, characteristics, or functions of a target concept, with the key term omitted. Students were required to recall and provide the appropriate term from memory based on the description. The 25-min duration was chosen to simulate the time pressure characteristic of CBT environments, thereby encouraging students to practice rapid retrieval of terminology under constrained conditions. A score of at least 45 correct answers (90%) was required to earn the bonus point assigned to each terminology test. This high threshold was intended to encourage a high level of recall accuracy and was consistent with institutional practice for formative assessments. The test items focused primarily on factual recall of key physiological terminology rather than higher-order application, in order to isolate the effect of repeated retrieval on terminology acquisition.

Terminology tests were administered eight times during the semester, typically at the conclusion of each lecture unit after students had been exposed to the relevant content and glossary. Tests were conducted in a classroom setting under invigilated conditions, and students were not allowed to use the glossary or other materials during the test. One bonus point was awarded for each test passed, with a maximum of eight bonus points added to the final course score. These bonus points were added to the total score derived from regular examinations and were intended as an extrinsic incentive to encourage consistent preparation and participation.

In addition to the terminology tests, three regular examinations were conducted during the semester, each scored on a 100-point scale (total possible score: 300 points). These examinations assessed broader physiological knowledge, including conceptual understanding and problem-solving, and were independent of the terminology test scores. The bonus points from the terminology tests were added to the cumulative examination score at the end of the course. To enhance transparency and allow readers to understand the structure and content of the educational materials used in this study, representative excerpts from the Dental Physiology Key Terminology Glossary and sample terminology test items are provided in the Supplementary Materials (Supplementary Files S1 and S2).

### 2.3. Survey Instrument

The questionnaire used in this study was developed specifically for this research to evaluate the unique components of the implemented terminology test system and its perceived impact on students’ learning experiences. The instrument was designed to capture multiple dimensions of student perceptions, including cognitive load, time burden, perceived usefulness, and motivational effects.

To ensure face validity and clarity of each item, the questionnaire underwent internal pilot testing and content validation. Four faculty members experienced in dental physiology education independently reviewed the draft items for relevance, comprehensiveness, and alignment with the educational objectives of the intervention. In addition, a small group of senior dental students who had already completed the physiology course were invited to comment on item wording, response options, and overall comprehensibility. Based on their feedback, minor phrasing adjustments were made to reduce ambiguity and improve readability before final administration. No items were removed during this process, and the final instrument retained its original conceptual structure.

An end-of-semester questionnaire was distributed to all students after completion of the eighth terminology test. The instrument comprised eight quantitative Likert-scale items and one open-ended question. The quantitative items assessed the following domains: (1) **Perceived test scope** (few, somewhat few, appropriate, somewhat excessive, excessive); (2) **Perceived test time** (short, somewhat short, appropriate, somewhat long, long); (3) **Perceived number of questions** (few, somewhat few, appropriate, somewhat excessive, excessive); (4) **Glossary’s impact on lecture understanding** (improved, somewhat improved, neither, hardly improved, did not improve); (5) **Motivational effect of bonus points** (enhanced, somewhat enhanced, neither, somewhat not enhanced, not enhanced); (6) **Glossary difficulty/clarity** (clear, somewhat clear, neither, somewhat difficult, difficult); (7) **Preparation time for each test** (<30 min, 30 min–1 h, 1–3 h, 3–5 h, >5 h); (8) **Frequency of regular glossary use outside tests** (never, 1–5 times, 6–10 times, 11–20 times, >20 times).

The open-ended item solicited participants’ free comments on the terminology tests and glossary, with particular attention to their perceived relevance to the Japanese National Dentist Examination and CBT-style assessments. Students were encouraged to describe both positive and negative aspects of the system, including suggestions for improvement, perceived alignment with examination content, and any unintended burdens or benefits. Responses were written in Japanese and later transcribed verbatim for analysis.

### 2.4. Data Analysis

Given the exploratory and descriptive nature of the study, analyses were limited to non-inferential statistics. Quantitative responses from the Likert-scale items were summarized using frequencies and percentages for each response category. All percentages were rounded to one decimal place where appropriate; therefore, totals may not equal exactly 100% due to rounding. No subgroup analyses (e.g., by gender or academic performance) were conducted, as the data were collected anonymously and not linked to individual demographic or academic records.

Qualitative responses from the open-ended question were analyzed thematically using an inductive approach. Thematic analysis was conducted following Braun and Clarke’s six-step framework [15]. First, two independent coders (faculty members with experience in dental education research) familiarized themselves with the data by reading all responses multiple times. Second, they generated initial codes by identifying meaningful units of text related to students’ perceptions of test burden, perceived usefulness, motivational effects, and relevance to national examinations. Coding was conducted separately for each coder to preserve independent interpretation.

Third, the coders collated codes into potential themes, grouping related codes that reflected broader patterns in the data (e.g., “enhanced exam readiness,” “time burden and stress,” “clarity and usability of glossary,” “suggestions for alignment with national examination”). Fourth, themes were reviewed and refined through iterative discussion, with attention to internal coherence and distinctiveness between themes. Fifth, themes were defined and named to capture their core essence and to facilitate clear reporting. Finally, representative quotations were selected to illustrate each theme in Section 3.

To enhance trustworthiness and analytic rigor, several strategies were employed. We maintained an audit trail documenting coding decisions, theme development, and consensus processes. Triangulation between coders was used to compare interpretations and identify potential biases. Consensus-based resolution procedures were employed to resolve disagreements, with both coders discussing divergent interpretations until agreement was reached. These procedures were designed to strengthen the credibility and dependability of the qualitative findings and to provide a transparent account of students’ perceptions of the terminology test system.

## 3. Results

### 3.1. Assessment of Test Structure (Scope, Time, and Number of Questions)

**Figure 1:**
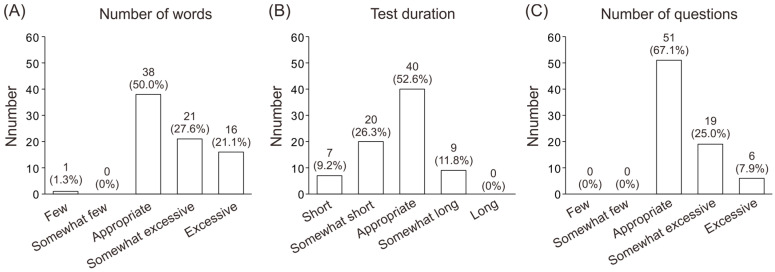
**Students’ perceptions of test structure.** (**A**) Perceived appropriateness of test scope (number of terms per unit). (**B**) Perceived appropriateness of test duration (25 min). (**C**) Perceived appropriateness of the number of questions (50 items per test). Data are presented as frequencies with percentages. Percentages may not sum to 100% due to rounding.

Survey responses indicated that students generally perceived the fundamental structure of the terminology tests as appropriate, although notable proportions reported areas of challenge. Regarding test scope (number of terms covered per unit), half of the respondents (50.0%, *n* = 38) rated the scope as appropriate. In contrast, 48.7% perceived the scope as somewhat excessive (*n* = 21) or excessive (*n* = 16), suggesting that the breadth of terminology required for each unit imposed a moderate cognitive burden (Figure 1A). This pattern aligns with the known difficulty of mastering large volumes of specialized physiological terminology in preclinical dental education.

For test duration, 52.6% (*n* = 40) considered the 25-min time limit appropriate, indicating that the majority felt able to complete the test within the allotted time. However, 35.6% (*n* = 27) reported that the time was short or somewhat short (Figure 1B), reflecting the time pressure inherent in rapid-recall assessments and suggesting that some students may have experienced difficulty retrieving terminology under constrained conditions.

The number of questions (50 items) received the highest level of agreement among all structural components, with 67.1% (*n* = 51) identifying it as appropriate (Figure 1C). This finding suggests that students were generally comfortable with the density of test items, even when the overall scope and time limit were perceived as demanding.

Taken together, these results indicate that the test structure was broadly acceptable to students, although the combination of broad terminology coverage and limited time may have contributed to perceived difficulty for some students.

### 3.2. Impact on Learning Outcomes (Understanding, Motivation, and Clarity)

**Figure 2:**
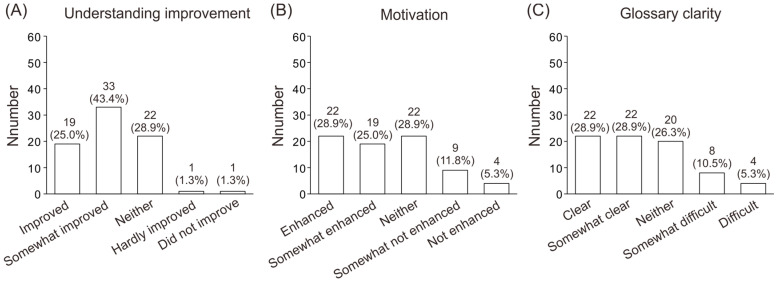
**Effects of the glossary and bonus point system on learning and motivation.** (**A**) Perceived impact of the glossary on lecture comprehension. (**B**) Perceived motivational effect of bonus points. (**C**) Perceived clarity of the glossary content.

Students reported positive perceptions regarding the glossary’s contribution to lecture comprehension. A total of 68.4% (*n* = 52) indicated that the glossary “significantly improved” or “somewhat improved” their understanding of physiological content, demonstrating that structured terminology support can enhance conceptual integration during lectures (Figure 2A). Only 2.6% (*n* = 2) reported little to no improvement, suggesting that the glossary was broadly effective across the cohort.

Motivational effects were more varied. The bonus point system was associated with increased motivation for 53.9% (*n* = 41) of students, who reported that it “enhanced” or “somewhat enhanced” their motivation to study (Figure 2B). Meanwhile, 28.9% (*n* = 22) reported neutral effects, and 17.1% (*n* = 13) indicated minimal motivational impact. These findings suggest that extrinsic incentives were beneficial for many students but not universally influential, reflecting individual differences in motivational drivers within demanding preclinical curricula.

Students also evaluated the clarity of glossary explanations. A majority (57.8%, *n* = 44) perceived the glossary as clear or somewhat clear (Figure 2C), indicating that the glossary’s structure—definitions, mechanistic explanations, and diagrams—was generally effective. However, the remaining students expressed neutral or negative perceptions, suggesting that some glossary entries may require refinement to better support comprehension.

Overall, these results demonstrate that the glossary contributed positively to learning outcomes, particularly in enhancing lecture comprehension, while motivational and clarity-related effects varied among individuals.

### 3.3. Study Habits and Usage Patterns

**Figure 3:**
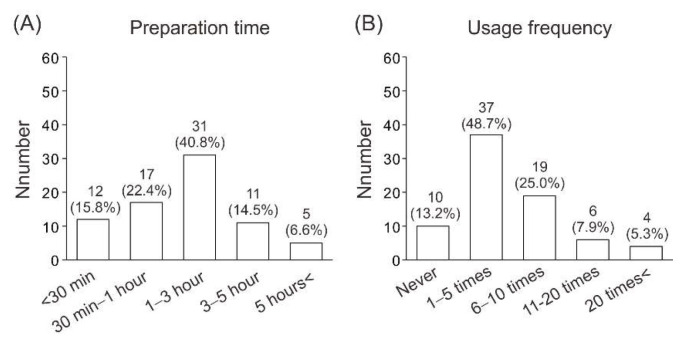
**Study behavior and glossary usage.** (**A**) Self-reported preparation time for each test cycle. (**B**) Frequency of glossary use during regular study.

Students reported diverse study habits and preparation times for the terminology tests. The largest proportion (40.8%, *n* = 31) spent 1–3 h preparing for each test, indicating moderate engagement with the material. Another 22.4% (*n* = 17) studied for 30 min to 1 h, and 15.8% (*n* = 12) spent less than 30 min, suggesting that a subset of students relied on rapid or minimal preparation (Figure 3A). Conversely, 21.1% (*n* = 16) devoted more than 3 h to preparation, reflecting substantial variability in study strategies and perceived difficulty.

Glossary usage patterns also varied. Nearly half of the students (48.7%, *n* = 37) reported using the glossary 1–5 times for routine study, while 38.2% (*n* = 29) used it six times or more (Figure 3B). These findings indicate that the glossary served not only as a test preparation tool but also as a supplementary resource for ongoing study throughout the semester.

To provide an integrated overview of the quantitative findings presented in Figure 1, Figure 2 and Figure 3, a summary table consolidating students’ perceptions of test structure, glossary usefulness, motivational effects, preparation time, and usage frequency is presented in Table 1. This table allows readers to review all key quantitative results in a single location and facilitates comparison across domains.

### 3.4. Qualitative Feedback from Open-Ended Responses

Twenty-nine open-ended responses were analyzed using inductive thematic analysis, resulting in three overarching themes that contextualize the quantitative findings and provide deeper insight into students’ experiences.

**Theme 1: Perceived learning benefits.** Students frequently described the terminology tests and glossary as beneficial for reinforcing lecture content and improving comprehension. Many noted that repeated exposure to key terms facilitated understanding of physiological mechanisms and supported preparation for practical sessions and regular examinations. “The glossary helped me understand the mechanisms more clearly.” “Reviewing the terms each week made the lectures easier to follow.”

**Theme 2: Motivational effects.** Several students reported that the bonus point system encouraged consistent study habits and helped them maintain engagement throughout the semester. The regular testing schedule also contributed to sustained preparation. “The bonus points motivated me to keep up with the material.” “Knowing there was a test every unit pushed me to study regularly.”

**Theme 3: Challenges and suggestions for improvement.** Students identified several challenges, including the large number of terms, scheduling conflicts with other examinations, and the high passing threshold. Suggestions for improvement included adjusting test timing, refining vocabulary selection, and adopting CBT-style multiple-choice formats to better reflect national examination conditions. “Memorizing so many terms during busy weeks was tough.” “A CBT-style multiple-choice format would make the test more realistic.”

These qualitative findings complement the quantitative results by illustrating how students experienced the terminology test system in practice, highlighting both its perceived benefits and areas for refinement.

## 4. Discussion

The implementation of key terminology tests in dental physiology education for second-year students represents a targeted approach to managing terminology overload within an examination-oriented curriculum. Designed to support preparation for the CBT format and the National Dentist Examination, this intervention integrates formative assessment into foundational learning [8,16]. From a programmatic assessment perspective, repeated low-stakes testing provides continuous feedback and promotes ongoing learning rather than episodic performance, aligning with assessment-for-learning models [17]. The present findings, based on both quantitative and qualitative data, suggest that combining periodic terminology tests with a glossary enhances lecture comprehension, encourages consistent study habits, and supports motivation, while also highlighting areas for refinement to better align with CBT requirements [16]. These findings highlight the potential of structured terminology-based interventions as a promising strategy for bridging foundational knowledge and examination readiness in dental education.

Students’ perceptions of test burden provide insight into workload management during the pre-clinical phase. Half of respondents rated the test scope as appropriate, indicating that approximately 65 terms per unit is a reasonable balance for physiology learning [5,18]. However, many perceived it as excessive, reflecting the high cognitive load required to both memorize and contextualize terminology related to orofacial functions assessed in the National Dentist Examination [19]. This aligns with cognitive load theory, which suggests that excessive information may hinder learning without adequate scaffolding [5,18]. Time allocation emerged as a related issue. Although most students found the 25-min duration appropriate, over one-third felt it was insufficient, suggesting a mismatch with the rapid recall demands of CBT environments [18]. In contrast, the number of questions was well accepted, indicating that adjustments to time rather than content volume may be beneficial [18]. Preparation time data further contextualize these findings. Most students reported studying 1–3 h per test cycle, representing a manageable level of engagement [20], while a smaller group studied more than 3 h, likely reflecting awareness of the importance of repeated exposure and spaced repetition for exam preparation [21,22]. These patterns align with previous evidence showing that low-stakes assessments can focus student effort and reduce stress [23,24]. These findings are consistent with previous studies in dental education demonstrating that low-stakes testing enhances student engagement and promotes efficient learning strategies [23,24].

The glossary was a key strength of the intervention, with most students reporting improved comprehension. This supports the value of supplementary resources that link terminology to clinical contexts using concise definitions and visual aids. From a theoretical perspective, the glossary likely reduced cognitive load, supporting deeper processing required for CBT-style questions [25]. Regular use of the glossary also suggests its role as a continuous learning tool, promoting self-directed learning prior to clinical training. Variability in perceived clarity may reflect differences in prior knowledge among students. Importantly, such structured exposure and repeated retrieval may help students adapt to the time-pressured and application-oriented nature of CBT.

Motivation was moderately enhanced by the bonus point system, consistent with self-determination theory, which indicates that structured external incentives can support engagement when appropriately implemented [26]. Qualitative responses suggested that transparent reward systems promoted consistent study behavior [26]. However, some students experienced limited motivational benefit, often associated with low preparation time and difficulty meeting thresholds. Introducing tiered incentive systems may improve equity while maintaining motivation [27].

Qualitative findings reinforced these results, highlighting perceived improved understanding and better integration of lecture content, and students’ perceived readiness for examinations. Students also recognized the role of repeated testing in supporting long-term retention, although this perception was not objectively measured in this study [8]. Challenges included limited testing time and scheduling conflicts, reflecting common constraints in dense pre-clinical curricula [28]. Students suggested improvements such as CBT-style questions, pre-unit testing, increased time allowances, and content prioritization [9,29].

This study has several limitations. It was conducted at a single institution, limiting generalizability. The absence of a control group and reliance on self-reported perceptions restrict the ability to draw conclusions about objective learning outcomes. Social desirability bias may have influenced responses. No longitudinal follow-up or performance data were collected, preventing evaluation of long-term retention or examination success. Future studies incorporating objective measures and multi-institutional designs are needed.

## 5. Conclusions

Students perceived that the key terminology tests and accompanying glossary enhanced comprehension, encouraged habitual study, and supported motivation. Although students perceived the terminology test system and glossary as useful, motivating, and supportive of lecture comprehension, the findings are based solely on post-intervention self-reported perceptions and do not provide objective evidence of improved knowledge retention or examination performance. Despite manageable perceived burdens and identifiable limitations, the system shows promise as a preparatory tool for both the CBT format and the National Dentist Examination. With iterative refinement focusing on time allocation, scheduling, and incentive structure, this intervention has the potential to serve as a scalable model for strengthening foundational physiological understanding and supporting perceived readiness for licensure examinations.

## Figures and Tables

**Table 1:** Summary of quantitative survey results for test structure, glossary utility, motivation, and study habits.

Survey Item	Response Category	*n* (%)
Test scope (number of words)	Appropriate	38 (50.0%)
	Somewhat excessive/Excessive	37 (48.7%)
Test duration (25 min)	Appropriate	40 (52.6%)
	Short/Somewhat short	27 (35.6%)
Number of questions (50 items)	Appropriate	51 (67.1%)
Glossary impact on lecture comprehension	Improved/Somewhat improved	52 (68.4%)
	Little or no improvement	2 (2.6%)
Motivational effect of bonus points	Enhanced/Somewhat enhanced	41 (53.9%)
	Neither	22 (28.9%)
	Minimal impact	13 (17.1%)
Glossary clarity	Clear/Somewhat clear	44 (57.8%)
Preparation time	<30 min	12 (15.8%)
	30 min–1 h	17 (22.4%)
	1–3 h	31 (40.8%)
	>3 h	16 (21.1%)
Glossary usage frequency	1–5 times	37 (48.7%)
	≥6 times	29 (38.2%)

## Data Availability

The data presented in this study are included in the article. Further inquiries can be directed to the corresponding author.

## Supplementary Materials

The following supporting information can be downloaded at: https://www.mdpi.com/article/10.3390/dj14070461/s1, Supplementary File S1: Sample Glossary Entries; Supplementary File S2: Sample Test Items.
