# Supplementary material for: Student Perceptions of Key Terminology Tests in Dental Physiology Education: A Single-Institution Evaluation
Source: Dent J (Basel). 2026 Jul 22;14(7):461. doi: 10.3390/dj14070461 (PMC13408308; doi:10.3390/dj14070461)
Supplement: Supplementary file 1 [file dentistry-14-00461-s001.zip › dentistry-4370322-supplementary.pdf]

Supplementary File S1. Sample Glossary Entries

Below are representative sample entries from the Dental Physiology Key Terminology Glossary used in the course. These examples illustrate the structure, level of detail, and educational purpose of the glossary provided to students.

Sample Glossary Entries

1. Central nervous system

Definition: A collective term referring to the brain and spinal cord.

Explanation: The central nervous system integrates sensory information, generates motor commands, and regulates vital functions. It works together with the peripheral nervous system to control the entire body.

2. Peripheral nervous system

Definition: The nervous system located outside the brain and spinal cord.

Explanation: It consists of the somatic nervous system (sensory and motor components) and the autonomic nervous system (sympathetic and parasympathetic divisions). It connects the CNS with organs and tissues throughout the body.

3. Myelinated nerve

Definition: A nerve fiber whose axon is covered by a myelin sheath.

Explanation: Myelin provides electrical insulation, enabling saltatory conduction and rapid transmission of action potentials.

4. Unmyelinated nerve

Definition: A nerve fiber without a myelin sheath.

Explanation: Conduction velocity is relatively slow. C fibers are representative unmyelinated fibers that transmit slow pain and temperature sensations.

5. Osmotic pressure

Definition: Pressure generated across a semipermeable membrane due to differences in solute concentration.

Explanation: Water moves from the side with a lower solute concentration to the side with a higher solute concentration. Osmotic pressure is essential for fluid balance, blood pressure regulation, and edema formation.

Supplementary File S2. Sample Test Items

Below are representative sample items from the terminology tests administered during the dental physiology course. Each item presents a definition or description, followed by a blank space for students to write the correct term.

Sample Fill-in-the-Blank Test Items

1. A division of the autonomic nervous system that becomes active during stress or when responding to the external environment. It exerts excitatory effects on the heart and inhibitory effects on gastrointestinal motility.

Answer: ______________________________

2. A division of the autonomic nervous system that becomes active during rest and recovery. It exerts inhibitory effects on the heart and enhances gastrointestinal motility.

Answer: ______________________________

3. A type of nerve fiber whose axon is covered by a myelin sheath. Action potentials propagate by saltatory conduction, resulting in rapid conduction velocity.

Answer: ______________________________

4. A type of nerve fiber without a myelin sheath. Conduction velocity is relatively slow, and C fibers are typical examples.

Answer: ______________________________

5. A specialized structure of myelinated axons where the myelin sheath is absent. Voltage-gated Na^+ channels are densely concentrated here, enabling saltatory conduction.

Answer: ______________________________
